# LONGITUDINAL RELATIONSHIPS BETWEEN RESOURCES, MOTIVATION, AND ENGAGEMENT IN COGNITIVELY DEMANDING ACTIVITIES

**DOI:** 10.1093/geroni/igz038.1131

**Published:** 2019-11-08

**Authors:** Claire Growney, Thomas M Hess

**Affiliations:** North Carolina State University, Raleigh, North Carolina, United States

## Abstract

Selective Engagement Theory (SET; Hess, 2014) suggests that decreases in personal resources and increases in the costs associated with activity engagement in old age negatively influence the motivation to engage in cognitively demanding activities. Here we explore these ideas longitudinally including a wide range of personal resources (cognitive ability, physical health, emotional health, and sensory functioning), with the expectation that emotional health might be a particularly important resource for older adults given its relative preservation with age. Young (n=125; age 19-42 at Time 1) and older adults (n=183; age 60-85 at Time 1) were tested from two to five times between years 2010 and 2016. Resources, motivation, and self-reported activity engagement (VLS Activity Questionnaire) were assessed at each time point. Using multilevel structural equation modeling, we found that changes in emotional health and sensory functioning predicted changes in motivation to engage in cognitively demanding activities. Additionally, increases in motivation predicted increases in engagement in cognitively demanding activities (e.g., technical, developmental), but decreases in less demanding activities (e.g., TV watching). Lastly, motivation partially mediated the relationships between emotional health and these activities, as well as between sensory functioning and engagement in technical activities. Results provide support for SET, demonstrating associations between changes in resources, motivation, and engagement in activities that are particularly demanding of cognitive resources, with the strength of these relationships being stronger in older than in young adults. Our results suggest that emotional resources may be particularly influential in determining the motivation for activity engagement in later life.

